# Lateralization of interictal epileptiform discharges differently impacts cognitive performance and behavior in late-onset epilepsy of unknown aetiology

**DOI:** 10.3389/fnagi.2026.1821590

**Published:** 2026-05-28

**Authors:** Giusy Bergamo, Mariana Fernandes, Gianluca Albanesi, Matteo Antonucci, Ilaria Barbaro, Beatrice Bertini, Flavia Cirillo, Valentina Corsi, Silvia Maio, Greta Testone, Nicola B. Mercuri, Diego Centonze, Claudio Liguori

**Affiliations:** 1 Department of Systems Medicine, University of Rome Tor Vergata, Rome, Italy; 2 Neurology Unit, University Hospital of Tor Vergata, Rome, Italy; 3 IRCSS Neuromed, Pozzilli, Italy

**Keywords:** delayed verbal memory, IEDs, late-onset epilepsy, MCI, neurodegeneration

## Abstract

**Background:**

Late-onset epilepsy of unknown aetiology (LOEU) is increasingly recognized as a multifactorial condition potentially linked to preclinical neurodegeneration. Despite its clinical relevance, the impact of lateralized interictal epileptiform discharges (IEDs) on cognitive and behavioral phenotypes remains poorly understood.

**Methods:**

We conducted an observational cross-sectional study of 62 LOEU patients (66.1 ± 7.3 years). All participants underwent neurological examination, electroencephalography, and a neuropsychological battery. Subjective sleep quality (Pittsburgh Sleep Quality Index), daytime sleepiness (Epworth Sleepiness Scale), depressive symptoms (Beck Depression Inventory-II), and quality of life (Quality of Life in Epilepsy Inventory-31) were also assessed. Patients were stratified based on IED lateralization (Left vs. Right).

**Results:**

IEDs were left-lateralized in 61.3% (*n* = 38) and right-lateralized in 38.7% (*n* = 24). A significant difference emerged only for delayed verbal recall (RAVLT-D), with right-lateralized patients performing worse (*p* = 0.043). No differences were found in other cognitive domains, sleep, mood, or quality of life.

**Conclusion:**

While right-sided IEDs were associated with lower RAVLT-D performance, this represents a nominally significant finding in the context of multiple domains tested without correction for multiple comparisons. These results may suggest a distributed pattern of cognitive vulnerability, possibly reflecting network dysfunction or inefficient interhemispheric integration. Findings should be interpreted with caution and considered exploratory. Given that RAVLT-D is a marker of hippocampal dysfunction and early neurodegeneration, these results support longitudinal monitoring to identify LOEU patients at higher risk of dementia, regardless of focal lateralization.

## Background

Epilepsy remains one of the most prevalent chronic neurological disorders worldwide, affecting over 50 million people with a characteristic bimodal incidence distribution that peaks in pediatric and elderly populations (Hirose, 2013). Notably, in adults over the age of 50, Late-Onset Epilepsy (LOE) shows a rising incidence, estimated at approximately 300 new cases per 100,000 person-years (Pervin et al., 2021; Zawar et al., 2025). Within this cohort, approximately 20% of cases lack an identifiable structural or metabolic aetiology, categorized as LOE of Unknown aetiology (LOEU) (Punia et al., 2025). Clinically, LOEU is predominantly characterized by focal manifestations—with or without bilateral tonic–clonic evolution—accounting for 61.9% of cases (Cao et al., 2025). A growing body of evidence suggests that preclinical neurodegenerative processes may play a pathogenic role in LOEU, as research by Hickman et al. (2023) (Hickman et al., 2023) indicates that neurodegeneration related to *β*-amyloid and tau deposits may precede seizure onset. Furthermore, cerebrospinal fluid biomarker studies have confirmed subclinical neurodegenerative signatures in LOEU patients (Fernandes et al., 2022), supporting the hypothesis that late-onset seizures may be an early clinical marker of an underlying dementing process. The cognitive trajectory of LOEU patients is a subject of intense research, with approximately 59% of patients exhibiting signs of Mild Cognitive Impairment (MCI) at the time of epilepsy onset (Nardi Cesarini et al., 2020). Longitudinal data are even more concerning, showing a 10-year cumulative dementia incidence of 22.2% in LOEU cohorts, with higher risk profiles identified in women and patients displaying interictal epileptiform discharges (IEDs) on baseline electroencephalography (EEG) (Ophir et al., 2021). Neuropsychological investigations have documented significant declines in global cognition (MMSE) and memory performance over 12-month periods, independent of anti-seizure medication (ASM) effects (Liguori et al., 2019). Compared to MCI subjects non-affected by epilepsy, LOEU-MCI patients demonstrate poorer performance in visuospatial abilities and executive functions, often presenting with a multi-domain MCI subtype (Nardi Cesarini et al., 2020). However, these profiles remain heterogeneous: 43.5% maintain normal cognitive performance, 39.1% exhibit focal deficits, and 17.4% show multi-domain impairment (DiFrancesco et al., 2021). EEG alterations appear central to these cognitive variations, as small-world network properties of baseline EEG, particularly within the delta and alpha bands, have been shown to predict cognitive decline (Costa et al., 2021). Complementary functional neuroimaging via 18F-Fluorodeoxyglucose Positron Emission Tomography (18F-*FDG PET)* often reveals temporal glucose hypometabolism, frequently ipsilateral to IEDs, or multifocal clusters extending to extra-temporal regions (DiFrancesco et al., 2021). Clinical factors such as sleep disturbances further complicate this relationship, as poor sleep quality has been associated with increased *β*-amyloid levels in LOEU patients, suggesting a bidirectional interaction between sleep fragmentation, epileptiform activity, and proteinopathy (Wang et al., 2025; Liguori et al., 2021). Despite these insights, the lack of standardized cognitive and electrophysiological characterizations limits clinical management. LOEU emerges as a multifactorial condition where seizures, cognitive decline, and neurodegeneration intersect.

Based on current evidence, LOEU appears to be a distributed network dysfunction rather than a purely focal deficit, although temporal lobe involvement remains a prominent clinical feature. While analysis of small-world properties and cortical sources reveals widespread multi-regional alterations (Nardi Cesarini et al., 2020; Costa et al., 2021), EEG and 18F-FDG PET data simultaneously confirm the persistence of localised IEDs and glucose hypometabolic brain clusters (DiFrancesco et al., 2021). In this context, the hypothesis emerges that LOE may represent an epiphenomenon of an underlying neurodegenerative disorder, reflecting a global network vulnerability rather than being its primary cause. On the hypothesis that IEDs may influence cognitive and behavioural profile of LOEU patients, the main objective of this study is to verify whether lateralization of IEDs drives distinct neuropsychological patterns (consistent with a classical localizationist model) or whether the observed profile reflects a broader network dysfunction independent of the focus, thereby supporting the hypothesis of an underlying neurodegenerative process integrating IEDs. Furthermore, the present study evaluates the influence of modulating factors, including sleep disorders, depressive symptoms, and quality of life, to provide a more comprehensive clinical framework for this population.

## Methods

### Study design

This observational cross-sectional study enrolled people with epilepsy over the age of 50 diagnosed with LOEU at the Neurology Unit of the University Hospital of Rome Tor Vergata. Following the diagnosis of LOEU or the reclassification of a previous diagnosis of LOE into LOEU at our tertiary epilepsy center, all participants underwent a standardized diagnostic workup comprising a neurological examination, EEG, and an extensive second-level neuropsychological battery, in accordance with routine clinical practice, supplemented by self-report questionnaires assessing sleep quality, depressive symptoms, and quality of life.

Inclusion criteria required a clinical diagnosis of epilepsy according to current international diagnostic criteria (Hickman et al., 2023), seizure onset after age 50, and the absence of both focal structural lesions on brain magnetic resonance imaging (MRI) and known metabolic, infectious, or inflammatory triggers. Conversely, exclusion criteria consisted of a confirmed diagnosis of neurodegenerative diseases, a history of acute or previous cerebrovascular events, and severe psychiatric disorders that could compromise the reliability of the assessments.

### Neuropsychological assessment

The neuropsychological evaluation employed the Mental Deterioration Battery (MDB) (Carlesimo et al., 1996) and the Rey-Osterrieth Complex Figure Test (Caffarra et al., 2002), a combination widely validated in Italian diagnostic centers. To assess verbal functions, learning and episodic memory were measured via Rey’s 15-word immediate and delayed recall (Rey, 1958; Bortolini et al., 1971), while executive functions and language were investigated through phonological verbal fluency (Borkowski et al., 1967) and sentence construction tests (Gainotti et al., 1976). Visuospatial skills and nonverbal reasoning were explored using Raven’s Progressive Matrices 47’ (Raven, 1947), immediate visual recognition (Caltagirone et al., 1979), and constructive praxis tests involving freehand and planned drawing (Gainotti et al., 1977). The Rey-Osterrieth Complex Figure Test further provided a detailed quantitative and qualitative analysis of visuospatial memory and planning during both copy and recall phases.

Subjective clinical measures included the Epworth Sleepiness Scale (ESS) (Johns, 1991) to identify excessive daytime sleepiness (total score > 10) and the Pittsburgh Sleep Quality Index (PSQI) (Buysse et al., 1989) to evaluate overall sleep quality (total score > 5).

Depressive symptoms were assessed using the Beck Depression Inventory-II (BDI-II) (Beck et al., 1996), and quality of life was evaluated in all patients using the Quality of Life in Epilepsy Questionnaire (QOLIE-31) (Cramer et al., 1998) to assess the subjective impact of the disorder on daily functioning.

### Electroencephalography

EEG recordings were conducted between 9:00 and 12:00 a.m. for 15 min with eyes closed, utilizing an 8-min baseline, 4 min of hyperventilation, and 2 min of intermittent light stimulation (ILS). Data were acquired via a Galileo EBNeuro 32-channel system at a 128 Hz sampling frequency, using 16 Ag-AgCl electrodes positioned according to the international 10–20 system (Fp1, Fp2, F3, F4, C3, C4, P3, P4, O1, O2, F7, F8, T3, T4, T5, T6), with FPz as ground and Oz as reference. Additional electrodes monitored ECG signals to facilitate artifact removal, with signal quality maintained through a 0.3-s time constant, 50 Hz low-pass, and 50 Hz notch filters.

EEGs were independently reviewed by two neurologists (CL and SM). EEG variables were extracted according to the “background activity” and “interictal findings” categories under the standardized computer-based organized reporting of EEG (SCORE) standard (Beniczky et al., 2017). The lateralization of IEDs was determined based on the main localization of the electrode sites where the peak negativity was observed (i.e., the location of maximum negativity). The initial annotations were jointly reviewed by two neurologists (CL and SM) to reach a consensus conclusion. Disagreement was mediated by a third reviewer (NBM) who did not participate in the first-round evaluation. Therefore, the lateralization of IEDs served as the primary criterion for classifying the study population into two subgroups: those with left-sided and those with right-sided abnormalities.

### Statistical analysis

Statistical analysis was performed using IBM SPSS Statistics (version 25.0). Descriptive data were expressed as mean ± standard deviation (SD) or median and interquartile range (IQR). Given the non-normal distribution of several variables (confirmed by the Shapiro–Wilk test), comparisons between patients with left and right-lateralized EEG abnormalities were conducted using the Mann–Whitney U test. Significant differences in neurocognitive performance, sleep parameters (PSQI, ESS), mood (BDI-II), and quality of life (QOLIE-31) were determined with a threshold of *p* < 0.05. Effect sizes were calculated as rank-biserial correlations (r _rb_) to estimate the magnitude of group differences.

## Results

A total of 62 patients with LOEU (56.5% male), with a mean age of 66.1 ± 7.3 years, were included in the study. The median of epilepsy duration was 17.0 months [4.3–64.8]. IEDs were left-lateralized in 38 patients (61.3%) and right-lateralized in 24 patients (38.7%). Comprehensive demographic and clinical characteristics are summarized in Table 1.

**Table 1:** Demographic and clinical characteristics of the study sample.

**Variable**	**Total sample (*N* = 62)**	**Left-IEDs (*n* = 38)**	**Right-IEDs (*n* = 24)**
**Demographic characteristics**			
Age (years)	66.1 ± 7.3	67.2 ± 7.1	64.5 ± 7.3
Education (years)	10.5 ± 3.9	11.0 ± 4.0	9.7 ± 3.7
Sex, *n* (%)			
Male	35 (56.5)	20 (52.6)	15 (62.5)
Female	27 (43.5)	18 (47.4)	9 (37.5)
Handedness, *n* (%)* Right-handed	57 (91.9)	36 (94.7)	21 (87.5)
Left-handed	2 (3.2)	1 (2.6)	1 (4.2)
Ambidextrous	1 (1.6)	0 (0.0)	1 (4.2)
Epilepsy characteristics			
Epilepsy duration (months)	17.0 [4.3-64.8]	27.5 [5.0-109.0]	11.5 [4.0-30.0]
Seizure type, *n* (%)			
Focal to bilateral tonic–clonic	33 (53.2)	20 (52.6)	13 (54.2)
Focal impaired awareness non-motor	9 (14.5)	7 (18.4)	2 (8.3)
Focal impaired awareness motor	4 (6.5)	1 (2.6)	3 (12.5)
Focal aware non-motor	15 (24.2)	9 (23.7)	6 (25.0)
Focal aware motor	1 (1.6)	1 (2.6)	0 (0.0)
Seizure frequency (last 28 days)	0.50 ± 1.17	0.34 ± 0.71	0.75 ± 1.65
Seizure freedom, *n* (%)	42 (67.7)	28 (73.7)	14 (58.3)
Treatment characteristics			
Monotherapy, *n* (%)	57 (91.9)	36 (94.7)	21 (87.5)
Polytherapy, *n* (%)	4 (6.5)	2 (5.3)	2 (8.3)
No treatment, *n* (%)	1 (1.6)	0 (0.0)	1 (4.2)
Drug Daily Dose (DDD)	0.83 ± 0.35	0.79 ± 0.36	0.90 ± 0.33
Valproate	12 (19.4)	7 (18.4)	5 (20.8)
Eslicarbazepine acetate	9 (14.5)	7 (18.4)	2 (8.3)
Zonisamide	3 (4.8)	2 (5.3)	1 (4.2)
Lamotrigine	2 (3.2)	2 (5.3)	0 (0.0)
Carbamazepine	2 (3.2)	1 (2.6)	1 (4.2)
Other†‡	10 (16.1)	4 (10.5)	6 (25.0)
EEG characteristics			
Left IEDs, *n* (%)	38 (61.3)	–	–
Right IEDs, *n* (%)	24 (38.7)	–	–

IEDs, interictal epileptiform discharges.

*Two cases had missing data on handedness; percentages were calculated on the total sample (*n* = 62).

†Percentages for individual antiseizure medications are not mutually exclusive, as some patients were receiving polytherapy.

‡Includes brivaracetam, oxcarbazepine, phenobarbital, perampanel, and pregabalin.

**Figure 1:**
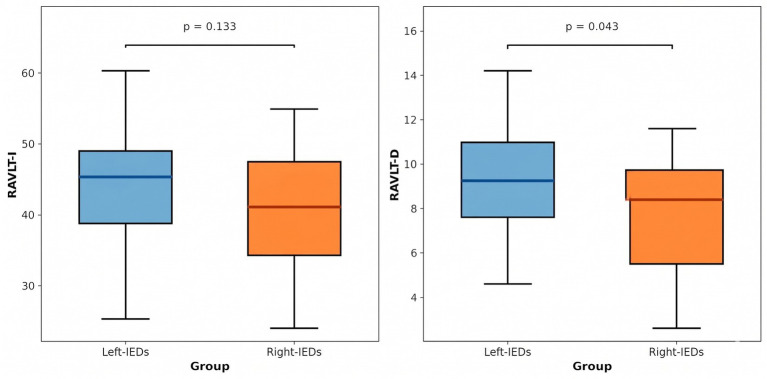
Boxplots showing RAVLT immediate recall (RAVLT-I) and delayed recall (RAVLT-D) scores in LOEU patients with left versus right-lateralized IEDs.

Regarding neuropsychological outcomes, a significant difference between groups was observed only for delayed verbal recall (RAVLT-D), with patients presenting with right lateralized IEDs performing significantly worse than those with left lateralized IEDs (*U* = 316, *p* = 0.043). In contrast, no significant inter-group difference was found for immediate verbal recall (RAVLT-I: *U* = 352, *p* = 0.133), phonological verbal fluency (*U* = 409, *p* = 0.502), semantic verbal fluency (*U* = 390, *p* = 0.340), or visual immediate memory (*U* = 414, *p* = 0.548). Similarly, performance was comparable between groups for freehand drawing copy (*U* = 376, *p* = 0.247), drawing copy with planning components (*U* = 419, *p* = 0.593), nonverbal reasoning assessed via Raven’s Coloured Progressive Matrices (*U* = 450 *p* = 0.931), and all indices of visuospatial memory (ROCF-C: *U* = 422, *p* = 0.829; ROCF-I: *U* = 428, *p* = 0.899; ROCF-D: *U* = 427, *p* = 0.882). Figure 1 shows the distribution of immediate and delayed verbal recall scores in the two groups of LOEU patients.

Furthermore, no significant difference was observed between subgroups regarding subjective sleep quality (*U* = 369, *p* = 0.207), daytime sleepiness (*U* = 344, *p* = 0.102), depressive symptoms (*U* = 373, *p* = 0.229), or quality of life (*U* = 400, *p* = 0.521). Detailed comparisons of cognitive and self-reported outcomes according to EEG lateralization are presented in Table 2.

**Table 2:** Comparison between LOEU subgroups based on the lateralization of interictal epileptiform discharges (IEDs).

Domains	**Left-IEDs (*n* = 38)**	**Right-IEDs (*n* = 24)**	^**Mann–**^ **Whitney U**	*p*-value	Effect size r_rb_
	**Median (P25–P75)**	**Median (P25–P75)**			
Cognitive domains					
RAVLT-I	45.3 (38.8–49.0)	41.1 (34.3–47.5)	352	0.133	–0.229
RAVLT-D	9.25 (7.60–11.0)	8.40 (5.50–9.72)	316	**0.043**	**–0.308**
Phonological Verbal Fluency	34.2 (28.9–39.2)	34.5 (24.1–38.7)	409	0.502	–0.103
Semantic Verbal Fluency	22.4 (19.8–24.6)	23.2 (21.1–24.9)	390	0.340	0.146
Visual Immediate Memory	20.6 (19.4–21.5)	20.9 (19.1–22.2)	414	0.548	0.092
Freehand Drawing Copy	10.1 (8.88–11.0)	10.4 (9.47–11.5)	376	0.247	0.177
Drawing Copy with Planning Components	65.3 (61.2–67.9)	64.7 (59.4–67.9)	419	0.593	–0.082
Raven’s Coloured Progressive Matrices	33.0 (29.3–34.9)	32.0 (29.5–35.5)	450	0.931	–0.014
ROCF-C	34.5 (32.5–36.1)	34.5 (33.0–36.4)	422	0.829	0.034
ROCF-I	14.3 (10.6–17.9)	14.5 (9.45–18.4)	428	0.899	–0.021
ROCF-D	14.4 (10.1–18.7)	14.5 (10.4–18.0)	427	0.882	–0.024
Sleep and Psychological domains					
PSQI	7.0 (4.0–9.0)	5.0 (2.0–9.0)	369	0.207	–0.191
ESS	3.5 (2.25–5.0)	4.0 (3.75–7.0)	344	0.102	0.247
QoLIE31	68.5 (58.4–79.1)	74.9 (57.4–84.6)	400	0.521	0.099
BDI-II	7.5 (3.25–14.0)	4.5 (2.0–10.0)	373	0.229	–0.183

RAVLT-I, Rey auditory verbal learning test—immediate recall; RAVLT-D, Rey auditory verbal learning test—delayed recall; ROCF-C, Rey–osterrieth complex figure test-copy; ROCF-I, Rey–osterrieth complex figure test—immediate recall; ROCF-D, Rey–osterrieth complex figure test—delayed recall; PSQI, Pittsburgh sleep quality index; ESS, Epworth sleepiness scale; QoLIE31, quality of life in epilepsy; BDI-II, beck depression inventory-II.

## Discussion

In this study, the impact of the lateralization of IEDs on the cognitive and behavioral profile of patients with LOEU was investigated. The primary finding of the present study was that patients with right-sided IEDs exhibit significantly diminished performance in delayed verbal memory (RAVLT-D) compared to those with left-sided IEDs (*p* = 0.043). This observation reinforces the hypothesis of heterogeneous neuropsychological profiles in LOEU (DiFrancesco et al., 2021) and challenges traditional material-specific models. While classical paradigms in temporal lobe epilepsy predict that left-sided foci primarily impair verbal memory whereas right-sided dysfunction affects visuospatial memory (Delaney et al., 1980; Pillon et al., 1999; Wagner et al., 2009), growing evidence suggests that verbal memory deficits can emerge regardless of lateralization of IEDs, reflecting distributed network dysfunction rather than strictly focal impairment (Saling, 2009; Giovagnoli et al., 2005). The observed “non-canonical” pattern in LOEU patients with right-sided IEDs may tentatively suggest inefficient functional reorganization or a disruption of long-term consolidation processes requiring effective interhemispheric integration; however, such interpretations remain speculative and warrant further investigation (Dupont et al., 2002; Ono et al., 2019). The RAVLT-D is a highly sensitive measure of delayed verbal episodic memory, specifically designed to assess the ability to retain and retrieve information over time by distinguishing between encoding, consolidation, and retrieval deficits. In epilepsy populations, RAVLT-D is a validated tool frequently applied to evaluate hippocampal dysfunction and consolidation failure (Ricci et al., 2022), with scores often correlating with clinical factors such as seizure focus, age, and the burden of anti-seizure medications (Subramaniam et al., 2020; Sarkis et al., 2018). Notably, in LOE cases, age above 50 years has been identified as a significant predictor of poor verbal memory outcomes on the RAVLT-D, reinforcing its clinical applicability and diagnostic relevance inthis specific demographic (Messinis et al., 2016). The medical literature on LOE often emphasizes clear hemispheric differences, frequently identifying the left hemisphere—and the left temporal lobe in particular—as more susceptible to seizure onset, structural changes (such as mesial temporal atrophy), and worse clinical outcomes, including higher seizure frequency and poorer prognosis (Ophir et al., 2021; Liguori et al., 2019). However, our findings in an LOEU cohort—supported by age-stratified normative data—suggest a more complex interaction where right-sided abnormalities significantly impact verbal consolidation. Consistently, the prevalence of left-sided IEDs was higher than right-sided IEDs in the population of patients with LOEU included. Impaired delayed recall is a hallmark of hippocampal dysfunction and is considered a robust marker for the early and prodromal stages of AD and MCI. Performance on the RAVLT-D is highly predictive of underlying neurodegenerative pathology and correlates strongly with hippocampal atrophy (Moradi et al., 2017); as such, it remains a key marker for the conversion from MCI to AD (Russo et al., 2017). Episodic memory, particularly delayed recall, is supported by a complex interplay between the medial temporal lobes, the default mode network, and frontal-parietal systems (Barnett et al., 2021; Westphal et al., 2017); thus, IEDs in the right hemisphere may interfere with the global efficiency of these widespread memory networks (Putcha et al., 2019). By identifying specific amnesia profiles, such as consolidation failure, researchers can better characterize the underlying pathology, whether it relates to selective hippocampal sclerosis or broader network failure (Ricci et al., 2022).

The reported difference in delayed verbal recall (RAVLT-D) is described as a nominally significant finding in the context of multiple cognitive domains tested without correction for multiple comparisons, and therefore should not be interpreted as evidence of a definitive lateralization-specific deficit. Accordingly, this finding should be interpreted with caution and considered exploratory.

While impaired delayed recall is widely recognized as a hallmark of hippocampal dysfunction and a robust marker of early and prodromal stages of AD and MCI, the present results do not allow for firm conclusions regarding underlying neurodegenerative processes. Instead, they highlight a potential signal that warrants further investigation in longitudinal and adequately powered studies.

The reported duration of epilepsy (median 17.0 months) reflects the time elapsed between the onset of seizure(s), as reported by the patient or their family members, and the time of evaluation at our tertiary epilepsy center. However, to ensure methodological consistency, all neuropsychological and EEG assessments were conducted in close temporal proximity to the evaluation at our center, in which—in some cases—reassessment of LOE into LOEU was done following the exclusion of etiopathological causes of epilepsy, during a dedicated visit that included also EEG recording and neuropsychological evaluation. This distinction explains the high variability: whilst the formal diagnosis of LOEU and study assessments were synchronized, the pre-diagnostic history of seizures or LOE diagnosis varied significantly within the cohort. As this study is exploratory and observational in nature, no correction for multiple comparisons was applied; however, the use of multiple statistical comparisons increases the nominal risk of Type I error. The moderate effect size observed for the primary significant finding further supports its biological relevance, which should nonetheless be regarded as hypothesis-generating and requires confirmation in larger prospective studies, in terms of spatial resolution for the precise localisation of IEDs sources. Although the literature confirms the high prevalence of abnormalities in the temporal lobe in patients with LOEU (DiFrancesco et al., 2021), standard EEG does not allow for a definitive distinction between generators in the lateral temporal regions and those in the mesial regions. This distinction appears crucial, as the involvement of mesial structures is often associated with specific limbic networks and different profiles of neurodegenerative vulnerability (Nardi Cesarini et al., 2020). Therefore, the lateralization of IEDs observed must be interpreted as the expression of a hemispheric or regional macro-dysfunction; future studies could clarify whether the anatomical-clinical precision of the epileptic focus further influences the pattern of cognitive decline compared to the distributed network dysfunction hypothesised here.

Furthermore, it is acknowledged that the routine EEGs recorded in this study were relatively brief, with a duration of approximately 15 min. It is noteworthy that this duration may fall below the minimum standards recently recommended for the identification of IEDs (Strigaro et al., 2024). Consequently, the frequency and presence of IEDs might have been underestimated in this study. Whilst this reflects standard clinical practice for initial screenings in many centres, future studies should consider prolonged EEG recordings to increase the diagnostic yield and provide a more comprehensive characterisation of the relationship between epileptiform discharges and cognitive dysfunction.

Finally, given that only two patients were left-handed and one was ambidextrous, the analysis was not adjusted for handedness. This parameter was considered unlikely to have influenced the overall results of the study.

In conclusion, the lack of significant differences between groups in other cognitive domains, sleep quality, mood, or quality of life supports the notion that cognitive decline in LOEU may be driven by global, subclinical neurodegenerative processes—such as *β*-amyloid or tau deposition—that transcend simple focal lateralization (Hickman et al., 2023; Fernandes et al., 2022; Liguori et al., 2021). These results underscore the necessity of moving beyond traditional lateralization theories toward a network-based understanding of LOEU. Longitudinal monitoring remains essential to identify patients at higher risk of progressing todementia, and future large-scale studies should incorporate assessments of Accelerated Long-Term (Ricci et al., 2024).

## Data Availability

The raw data supporting the conclusions of this article will be made available by the authors, without undue reservation.
